# Letter to the Editor-Extensive aortic thrombosis in a patient with diabetes mellitus and COVID-19

**DOI:** 10.1097/j.pbj.0000000000000156

**Published:** 2022-09-09

**Authors:** Catarina Cidade-Rodrigues, Pedro Palma, Rogério Ruas, Rita Ferraz

**Affiliations:** a Resident, Endocrinology Department, Centro Hospitalar do Tâmega e Sousa, Avenida do Hospital Padre Américo 210, 4564-007 Guilhufe, Penafiel, Portugal,; b Attending Physician, Infectious Diseases Unit, Centro Hospitalar do Tâmega e Sousa, Avenida do Hospital Padre Américo 210, 4564-007 Guilhufe, Penafiel, Portugal

**Keywords:** @#@, @#@, @#@, @#@, @#@, @#@, @#@, @#@, @#@, @#@

To the Editor,

We present a case of a 77-year-old male with diabetes mellitus (DM) admitted with severe acute respiratory syndrome corona-virus 2 (SARS-CoV2) pneumonia who further developed new-onset flaccid paraplegia of the lower limbs and signs of limb ischemia. His D-dimer level was 51,236ng/ml and computed tomography angiography showed extensive thrombosis (11 cm) of the infra-renal aortic segment, common, internal and external iliac arteries, and both femoral arteries. Symptomatic and palliative treatment was instituted and the patient died shortly after. This severe complication was assumed to be secondary to the synergic prothrombotic and hypercoagulable states inherent to both SARS-CoV-2 infection and DM.

## Introduction

Coronavirus disease 2019 (COVID-19) is associated with susceptibility to systemic thromboembolic complications and diabetes mellitus (DM) is a risk factor promoting severity of the disease and unfavorable prognosis.^1^ Scarce data exists concerning the prevalence of thrombotic complications in diabetic patients infected with severe acute respiratory syndrome coronavirus 2 (SARS-CoV-2). Although venous thromboembolism has already beenextensivelyreportedininfectedpatients,^2^ arterialthrombosis is now beginning to be noticed and reported in the literature, occurring in about 4% of critically ill COVID-19 patients with a mortality rate of 20%.^3^ Anatomically, these events showed a wide range of affected areas: limb arteries (39%), cerebral arteries (24%), great vessels [aorta, common iliac, common carotid, and brachiocephalic trunk (19%)], coronary arteries (9%), and superior mesenteric artery (8%).^3^ We describe a case of a patient admitted for SARS-CoV-2 infection, who, during hospitalization, developed an uncommon and extensive aortic thrombosis with a peculiar clinical presentation.

## Case report

**Figure 1.**
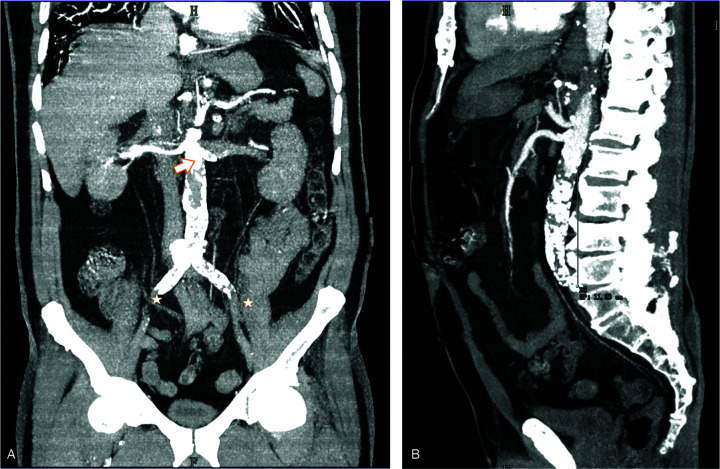
CT angiography. (A) Anterior MIP shows the thrombus extending below the renal arteries (arrow) and occlusion of common lilac arteries (stars). (B) Sagittal MIP image shows an abdominal aorta thrombosis with a longitudinal length of 11 cm. CT = computed tomography; MIP = maximum intensity projection.

A 77-year-old male patient presented to the emergency department with symptoms of exertional dyspnea and wheezing for a week. Past medical history included type 2 DM, hypertension, and dyslipidemia. Microvascular organ damage included peripheral diabetic neuropathy and macrovascular complications included ischemic heart disease. He had no cough, fever, malaise, or other complaints. He was admitted to the Infectious Diseases department with SARS-CoV-2 pneumonia with hypoxemic respiratory failure (PaO_2_/FiO_2_ ratio of 157). His blood work upon admission showed a normal prothrombin time and D-dimers 3462ng/ml. He was started on intravenous dexamethasone 6 mg id and prophylactic-dose enoxaparin. He was evolving well until day 5 of admission (day 14 of respiratory symptoms) when his respiratory distress worsened and he presented with extreme tiredness, lumbar back pain, new-onset lower limb paraparesis, and urinary retention prompting the introduction of a urinary catheter. He was hypertensive, tachycardic, tachypneic with peripheral oxygen saturation of 96% on a 40% Venturi mask. He had no fever. In the physical examination, there was flaccid paraplegia of the lower limbs, foot drop sign bilaterally, associated with signs of ischemia: pale and cold skin in both legs, thigh cyanosis, the absence of femoral, popliteal, and foot dorsal pulses, and unmeasurable arterial pressure. No signs of meningeal or cranial nerve affection were present. Arterial blood gas revealed a PaO2/FiO2 ratio of 207. Electrocardiography showed sinus rhythm tachycardia. His blood work on that day showed D-dimers 51,236ng/ml, high-sensitivity troponin 33,665.9pg/ml, myoglobin above 3948ng/ ml, hemoglobin 12.8 g/dl, and serum creatinine 1.68 mg/dl. Abdominal and pelvic computed tomography angiography (Fig. 1) showed the absence of opacification of the infra-renal aortic segment, with a longitudinal thrombosis of 11 cm, the absence of opacification of common, internal, and external iliac arteries, as well as femoral arteries bilaterally. Diagnosis of acute irreversible lower limb ischemia due to extensive infra-renal aortic thrombosis was made. In a multidisciplinary team meeting, it was decided that there was no indication to perform vascular surgery, thus symptomatic and palliative treatment was instituted. The patient died the following day. This severe complication was assumed to be secondary to the synergic prothrombotic and hypercoagulable states inherent to both SARS-CoV-2 infection and DM.

## Discussion

As this case highlights, acute aortic occlusion occurs with sudden clinical presentation and potentially catastrophic consequences, showing a mortality rate ranging from 34% up to 62%.^4,5^ In this case, it presented as a lower limb paralysis secondary to spinal cord ischemia. To the present date, there have only been a few reports of COVID-19 patients presenting with this kind of thrombotic complication involving the abdominal aorta and none showed such an extensive thrombus occluding the infrarenal aorta, iliac, and femoral arteries bilaterally. Twelve reports so far in the literature have indicated an aortoiliac location of thrombosis.^6-12^ One report, similarly to ours, retrospectively emphasized lower back pain as the first sign of aortoiliac arterial occlusion.^2^ In our patient, small embolic thrombi occluded Adamkiewicz artery and subsequently the anterior spinal artery, responsible for the supply of the lower two-thirds of the spinal cord, thus generating a predominantly motor paralysis. The vascular occlusion of the aorto-iliac-femoral regions was implicated in the lower limb irreversible ischemia. The synergic hypercoagulable effect of SARS-CoV-2 infection and DM, as well as other atherosclerotic risk factors, culminated in this catastrophic complication. There is a strong association between D-dimer levels and the prognosis of SARS-CoV-2 infection^13,14^ and patients with DM are more likely to develop a hypercoagulable state during infection.^15^ Prophylactic doses of low molecular weight heparin (LMWH) are currently recommended for all COVID-19 patients who need hospitalization.^16^ The most appropriate daily dose for these patients is yet to be defined, since the occurrence of thrombotic events has been reported in patients under prophylactic doses of LMWH, suggesting that this regimen may not be enough in some cases.^16,17^ Some panels, such as the International Society on Thrombosis and Haemostasis and the Anticoagulation Forum, accept dose-escalation either empirically or based on increasing D-dimer values.^18^ This case report reflects the importance of thrombotic prophylaxis in patients infected with SARS-CoV2, especially those with DM and multiple atherosclerotic risk factors.

## References

[1] Wijaya I. Andhika R. Huang I. Hypercoagulable state in COVID-19 with diabetes mellitus and obesity: is therapeutic-dose or higher-dose anticoagulant thromboprophylaxis necessary? Diabetes Metab Syndr. 2020;14 5:1241–1242. 10.1016/j.dsx.2020.07.015 PMC7362820 32683322

[2] Naudin I. Long A. Michel C. Devigne B. Millon A. Della-Schiava N. Acute aortoiliac occlusion in a patient with novel coronavirus disease-2019. J Vasc Surg. 2020. 10.1016/j.jvs.2020.10.018 PMC7568051 33075454

[3] Cheruiyot I. Kipkorir V. Ngure B. Misiani M. Munguti J. Ogeng’o J. Arterial thrombosis in coronavirus disease 2019 patients: a rapid systematic review. Ann Vasc Surg. 2020;70:273–281. 32866574 10.1016/j.avsg.2020.08.087 PMC7453204

[4] Dossa C.D. Shepard A.D. Reddy D.J. Acute aortic occlusion. A 40-year experience. Arch Surg. 1994;129 6:603–607. discussion 7–8. 8204034 10.1001/archsurg.1994.01420300041006

[5] Babu S.C. Shah P.M. Nitahara J. Acute aortic occlusion—factors that influence outcome. J Vasc Surg. 1995;21 4:567–572. discussion 73–75. 7707562 10.1016/s0741-5214(95)70188-5

[6] Wengerter S.P. Wengerter K.R. Masoudpoor H. Acute aortoiliac and infrainguinal arterial thrombotic events in four patients diagnosed with the novel coronavirus 2019. J Vasc Surg Cases Innov Tech. 2020;6 4:698–702. 33102991 10.1016/j.jvscit.2020.09.011 PMC7571440

[7] Baeza C. González A. Torres P. Pizzamiglio M. Arribas A. Aparicio C. Acute aortic thrombosis in COVID-19. J Vasc Surg Cases Innov Tech. 2020;6 3:483–486. 32838094 10.1016/j.jvscit.2020.06.013 PMC7366084

[8] Woehl B. Lawson B. Jambert L. Tousch J. Ghassani A. Hamade A. 4 Cases of aortic thrombosis in patients with COVID-19. JACC Case Rep. 2020;2 9:1397–1401. 32835285 10.1016/j.jaccas.2020.06.003 PMC7279760

[9] Gomez-Arbelaez D. Ibarra-Sanchez G. Garcia-Gutierrez A. Comanges-Yeboles A. Ansuategui-Vicente M. Gonzalez-Fajardo J.A. COVID-19-related aortic thrombosis: a report of four cases. Ann Vasc Surg. 2020;67:10–13. 32474145 10.1016/j.avsg.2020.05.031 PMC7256515

[10] Vulliamy P. Jacob S. Davenport R.A. Acute aorto-iliac and mesenteric arterial thromboses as presenting features ofCOVID-19. Br JHaematol. 2020;189 6:1053–1054. 32353183 10.1111/bjh.16760 PMC7267618

[11] Lari E. Lari A. AlQinai S. Severe ischemic complications in Covid-19—a case series. Int J Surg Case Rep. 2020;75:131–135. 32934901 10.1016/j.ijscr.2020.09.009 PMC7482595

[12] Katchanov J. Kalisch J. Herzing W. Extensive aortic thrombosis in a patient with COVID-19. Ann Emerg Med. 2020;76 3:373–374. 32828336 10.1016/j.annemergmed.2020.04.044 PMC7196387

[13] Zhang L. Yan X. Fan Q. D-dimer levels on admission to predict inhospital mortality in patients with Covid-19. J Thromb Haemost. 2020;18 6:1324–1329. 32306492 10.1111/jth.14859 PMC7264730

[14] Lippi G. Favaloro E.J. D-dimer is associated with severity of coronavirus disease 2019: a pooled analysis. Thromb Haemost. 2020;120 5:876–878. 32246450 10.1055/s-0040-1709650 PMC7295300

[15] Wang Z. Du Z. Zhu F. Glycosylated hemoglobin is associated with systemic inflammation, hypercoagulability, and prognosis of COVID-19 patients. Diabetes Res Clin Pract. 2020;164:108214. 32416121 10.1016/j.diabres.2020.108214 PMC7233217

[16] Bikdeli B. Madhavan M.V. Jimenez D. COVID-19 and thrombotic or thromboembolic disease: implications for prevention, antithrombotic therapy, and follow-up: JACC state-of-the-art review. J Am Coll Cardiol. 2020;75 23:2950–2973. 32311448 10.1016/j.jacc.2020.04.031 PMC7164881

[17] Klok F.A. Kruip M. van der Meer N.J.M. Incidence of thrombotic complications in critically ill ICU patients with COVID-19. Thromb Res. 2020;191:145–147. 32291094 10.1016/j.thromres.2020.04.013 PMC7146714

[18] Flaczyk A. Rosovsky R.P. Reed C.T. Bankhead-Kendall B.K. Bittner E.A. Chang M.G. Comparison of published guidelines for management of coagulopathy and thrombosis in critically ill patients with COVID-19: implications for clinical practice and future investigations. Critical Care (London, England). 2020;24 1:559. 10.1186/s13054-020-03273-y PMC7492793 32938471

